# Thyroid disorders as predictors of cemiplimab efficacy in recurrent/metastatic cervical cancer: real-world evidence from Poland

**DOI:** 10.3389/fimmu.2025.1604826

**Published:** 2025-06-20

**Authors:** Renata Pacholczak-Madej, Maja Lisik-Habib, Radosław Mądry, Monika Szarszewska, Zuzanna Borysiewicz, Katarzyna Gabalewicz, Ewa Iwańska, Wiktor Szatkowski, Mirosława Puskulluoglu, Jerzy Jakubowicz, Paweł Blecharz

**Affiliations:** ^1^ Department of Gynecological Oncology, Maria Sklodowska-Curie National Research Institute of Oncology, Krakow, Poland; ^2^ Department of Anatomy, Jagiellonian University, Medical College, Krakow, Poland; ^3^ Department of Proliferative Diseases, Copernicus Memorial Hospital in Lodz Comprehensive Cancer Center and Traumatology, Łódź, Poland; ^4^ Klinika Ginekologii Onkologicznej Uniwersytetu Medycznego w Poznaniu, Poznan, Poland; ^5^ Department of Oncology and Chemotherapy, Provincial Integrated Hospital, Elbląg, Poland; ^6^ Lower Silesian Oncology, Pulmonology and Hematology Center, Wrocław, Poland; ^7^ Department of Clinical Oncology, Maria Sklodowska-Curie National Research Institute of Oncology, Krakow, Poland

**Keywords:** cemiplimab, uterine cervical neoplasms, thyroid diseases, immune checkpoint inhibitors, immune-related adverse events

## Abstract

**Introduction:**

Immune checkpoint inhibitors have improved survival in patients with recurrent or metastatic cervical cancer (r/mCC), yet reliable predictors of treatment efficacy remain undefined. Immune-related adverse events (irAEs) have been suggested as potential predictors of response, but evidence in cervical cancer is limited.

**Methods:**

We conducted an ambispective, multicenter observational study of 37 patients with r/mCC treated with cemiplimab within Poland’s national rescue access program. Baseline characteristics, treatment outcomes, and irAEs were analyzed. Survival outcomes were estimated using the Kaplan–Meier method and compared using Cox proportional hazards models. A p-value of <0.05 was considered statistically significant.

**Results:**

After a median follow-up of 9.2 months, 17 episodes of irAEs were reported in 40.5% of patients (n=15), with thyroid disorders being the most common (n=11, 64.7%). Patients who developed ir-thyroid disorders had significantly longer progression-free survival (hazard ratio [HR]=0.2; 95% confidence interval [CI]: 0.07–0.6, p=0.004) and overall survival (HR=0.2; 95% CI: 0.05–0.9; p=0.04) compared to those without such events. Moreover, the objective response rate was notably higher in this group (45.5% versus 11.5%, p=0.04). Most irAEs were mild and manageable, with a median time to onset of two months after cemiplimab initiation.

**Conclusions:**

Ir-thyroid disorders may indicate enhanced immune activation and represent a potential surrogate of cemiplimab efficacy in r/mCC, although validation in larger cohorts is required.

## 1 Introduction

Cervical cancer remains a major contributor to cancer-related mortality among women worldwide. Significant advances in the management of early-stage cervical cancer—including fertility-sparing surgery, sentinel lymph node biopsy, and tailored adjuvant therapies—have contributed to improved oncologic outcomes and quality of life (1). Despite recent therapeutic advances, recurrent and metastatic cervical cancer (r/mCC) remains a major clinical challenge with limited long-term survival. Novel treatment strategies, including immune checkpoint inhibitors (ICIs), DNA damage repair inhibitors, and antibody–drug conjugates, are actively being investigated and hold promise for improving outcomes in this hard-to-treat population (2).

Eastern European countries, including Poland, report disproportionately high incidence rates of cervical cancer, with Poland registering 18.9 cases per 100,000 women annually, compared to the European average of 2.8 per 100,000 (3). Although the incidence has shown a gradual decline, cervical cancer mortality in Poland remains more than twice the European average (10.5 vs. 5.3 per 100,000 women) (3). This unfavorable outcome is primarily attributed to suboptimal uptake of human papillomavirus (HPV) vaccination, only introduced as a national program in 2023, low participation in cytological screening (<25%) (4, 5), and delayed access to innovative systemic therapies.

Cemiplimab is a monoclonal antibody targeting programmed cell death protein 1 (PD-1) that has demonstrated efficacy in patients with r/mCC treated in later lines of therapy and without prior exposure to ICIs. In the phase III EMPOWER-Cervical 1 trial (6), cemiplimab significantly reduced the risk of death by 30% compared to standard chemotherapy (hazard ratio [HR] for overall survival [OS]: 0.69; 95% confidence interval [CI]: 0.56–0.84) and lowered the risk of disease progression by 25% (HR for progression-free survival [PFS]: 0.75; 95% CI: 0.63–0.89). These benefits were associated with a moderate extension in median OS (12.0 vs. 8.5 months) and comparable median PFS (2.8 vs. 2.9 months). Programmed death-ligand 1 (PD-L1) expression was quantified as the Combined Positive Score (CPS- defined in the **Supplementary Materials**) and retrospectively assessed in archival tumor samples in approximately one-third of the study population; no survival advantage was observed in patients with CPS <1%. The results of this pivotal trial led to the approval of cemiplimab by the European Medicines Agency (EMA) for use irrespective of PD-L1 status (7). In Poland, cemiplimab has been reimbursed as a monotherapy for later-line treatment of r/mCC since January 2025. Before this, access to cemiplimab was limited to participation in clinical trials or through a rescue access program, which required approval by regional health authorities and was available exclusively in designated reference oncology centers, contingent upon the absence of other viable treatment options.

Given the modest clinical benefit of cemiplimab in unselected populations and ongoing uncertainties regarding the predictive value of molecular biomarkers such as PD-L1 expression, the identification of reliable prognostic and predictive markers remains crucial for optimal patient selection. Evidence regarding the clinical relevance of immune-related adverse events (irAEs) in this context is limited. While retrospective studies in other tumor types have suggested that the development of irAEs may serve as a surrogate marker of ICIs efficacy (8–10), data specific to r/mCC are sparse.

Therefore, this study aimed to explore the potential association between the occurrence of irAEs and clinical outcomes in patients with r/mCC treated with cemiplimab within a rescue access program in Poland.

## 2 Methods

### 2.1 Study design

This ambispective, multicenter real-world study was conducted in five Polish reference oncology centers after approval from the Bioethics Committee of the Maria Sklodowska-Curie National Research Institute of Oncology Branch Warsaw (approval number 93/2024, dated November 21, 2024). It included 37 adult patients diagnosed with r/mCC who had progressed after platinum-based chemotherapy and were treated under a rescue access program. Patients received cemiplimab (350 mg intravenously every three weeks) between October 1, 2022 and January 31, 2025. Baseline patient characteristics (including CPS and HPV status, when available) were collected retrospectively, whereas treatment outcomes and safety data were assessed prospectively with the data cut-off on March 1, 2025. The study adhered to the European Society for Medical Oncology (ESMO) Good Reporting of Outcomes in Real-World Evidence Studies (GROW) (11) guidelines, with details regarding its implementation and comprehensive methodology description provided in the **Supplementary Materials**.

### 2.2 Population

Eligibility criteria aligned with the pivotal clinical trial (6) and the summary of product characteristics (7). The study population was defined as follows:

Inclusion criteria:

- Histologically confirmed diagnosis of r/mCC, including:

 ○ Squamous cell carcinoma

 ○ Adenocarcinoma

 ○ Adenosquamous carcinoma

 ○ Clear cell carcinoma

- Local pathological verification of the diagnosis
- Measurable disease according to Response Evaluation Criteria in Solid Tumors version 1.1 (RECIST v1.1) guidelines (12).
- Eastern Cooperative Oncology Group (ECOG) performance status 0–2
- Adequate renal, hepatic, and bone marrow function
- No prior treatment with ICIs
- Inclusion irrespective of PD-L1 expression status

Exclusion criteria:

- Previous treatment with ICIs
- Active or recent autoimmune disease
- Ongoing immunosuppressive glucocorticoid therapy exceeding 10 mg of prednisone daily (or equivalent) within four weeks prior to cemiplimab initiation
- Active infections requiring systemic treatment (bacterial, viral, fungal, or mycobacterial)
- ECOG performance status >2
- Uncontrolled comorbidities that could interfere with treatment or study assessments
- Lack of informed consent or inability to cooperate with study staff

### 2.3 Objectives of the study

The primary objective of the study was to assess the safety profile of cemiplimab by characterizing the incidence, type, and severity of irAEs, graded according to the Common Terminology Criteria for Adverse Events (CTCAE) version 5.0 (13), and to evaluate their association with survival outcomes, specifically PFS and OS.

The secondary objective was to determine treatment efficacy, including PFS, OS, overall response rate (ORR), and disease control rate (DCR). Tumor response was assessed based on computed tomography (CT) scans of the chest, abdomen, and pelvis, performed every 12 weeks or earlier if clinically indicated. Radiological evaluations were conducted according to the RECIST v1.1 guidelines (12). Detailed definitions are provided in the **Supplementary Materials**.

### 2.4 Safety assessments

Adverse events (AEs) were evaluated according to the CTCAE version 5.0 (13). IrAEs were classified as toxicities of immune origin, recognized either by clinical evaluation or by the requirement for immunosuppressive intervention, in line with ESMO guidelines (14). The management of irAEs was conducted following ESMO recommendations (14).

In addition, all patients had a basic metabolic panel performed at baseline and before each treatment cycle, with routine measurement of thyroid-stimulating hormone (TSH) levels. Free thyroxine (T4) and 3,3′,5-triiodo-L-thyronine (T3) levels were evaluated when clinically indicated. Immune-related (ir-) thyroid disorders included three conditions that often transitioned from one to another, such as primary hypothyroidism, primary hyperthyroidism, and thyroiditis (definitions provided in **Supplementary Materials**).

### 2.5 Follow-up period

Patients were followed prospectively throughout cemiplimab treatment, with clinical evaluations performed at each drug administration every 3 weeks or earlier in cases where toxicity required medical assessment. Radiological assessments with CT scans of the chest, abdomen, and pelvis were scheduled every 12 weeks or earlier if clinically indicated based on the patient’s condition or symptom progression. Follow-up continued until disease progression, death, or data cut-off, whichever occurred first.

### 2.6 Statistical analysis

All statistical analyses were conducted using PS Imago Pro 9 software (based on SPSS). Group comparisons for categorical variables were performed using the chi-square test or Fisher’s exact test, as appropriate. PFS and OS were estimated using the Kaplan-Meier method, with survival curves compared by log-rank tests. Additionally, Cox proportional hazards regression models were applied to explore associations between selected variables and survival outcomes. A p-value of less than 0.05 was considered statistically significant.

## 3 Results

### 3.1 Baseline characteristics

**Table 1** presents the baseline characteristics of the study cohort. Squamous cell carcinoma was the predominant histological subtype, observed in 70.3% of patients (n=26), whereas HPV status was unavailable for most cases. At the time of initial diagnosis, 18.9% of patients (n=7) presented with primary metastatic disease, while the remaining individuals underwent radical treatment, most commonly definitive radiochemotherapy (n=27, 79.4%). In the first line setting for r/mCC, 52.8% of patients (n=19) received platinum-based regimens containing bevacizumab. Notably, 43.2% of the cohort (n=16) had been heavily pretreated and received two or more prior systemic therapies before cemiplimab initiation. CPS was available in approximately one-third of patients, with almost all of them showing CPS ≥1 (n=8, 21.6%). The most frequent sites of metastases included non-regional lymph nodes (n=20, 57.1%), lungs (n=15, 42.9%), and bones (n=10, 28.6%).

**Table 1:** Baseline clinical characteristics of the enrolled patients (n=37).

Age		58.5 (44-65.8)
Body-Mass Index (kg/m^2^)		23.7 (20.7-28.3)
Performance status, n(%)	0	5 (13.5)
	1	23 (62.2)
	2	9 (24.3)
FIGO stage at primary diagnosis, n(%)	I	5 (13.5)
	II	13 (35.1)
	III	11 (29.7)
	IV	7 (18.9)
	No data	1 (2.7)
Histology, n(%)	Squamous cell carcinoma	26 (70.3)
	Adenocarcinoma	10 (27)
	Clear cell	1 (2.7)
HPV status	Positive	3 (8.1)
	Negative	1 (2.7)
	Unknown	33 (89.2)
Combine Positive Score, n(%)	<1	1 (2.7)
	≥1	8 (21.6)
	Unknown	28 (75.7)
Number of previous lines of chemotherapy before cemiplimab initiation	1	21 (56.8)
	2	11 (29.7)
	3	4 (10.8)
	4	1 (2.7)

Categorical variables are presented as numbers (percentages), and continuous variables are presented as medians and interquartile ranges.

FIGO, International Federation of Gynecology and Obstetrics; HPV, human papillomavirus; n- number.

### 3.2 Safety

A total of 17 irAEs episodes were reported in 40.5% of patients (n=15). The most frequently observed irAEs were ir-thyroid disorders (n=11, 64.7%), followed by hepatotoxicity (n=3, 17.6%), infusion-related reaction (n=1, 5.9%), rheumatic toxicity (manifesting as musculoskeletal pain; n=1, 5.9%), and diarrhea (n=1, 5.9%). The majority of irAEs were of mild to moderate intensity, with 94.1% classified as grade (G) 1–2 according to CTCAE v5.0. One case (5.9%) of G3 hepatotoxicity was reported; no G4 or G5 events were observed. The median time to irAEs onset was 2 months (interquartile ranges [IQR]: 1–3 months) from treatment initiation. Systemic corticosteroids were administered in two patients at a median prednisone-equivalent dose of 1 mg/kg/day. No escalation to additional immunosuppressive therapy was required. Treatment discontinuation due to hepatotoxicity occurred in one patient (5.9%).

Patients who experienced irAEs demonstrated significantly improved PFS compared to those without irAEs. In the irAE-group, median PFS was not reached (NR; 95% CI: NR–NR), whereas in the non-irAE group, it was 3.3 months (95% CI: 1.1–5.6) (p=0.002). The occurrence of irAEs was associated with a 70% reduction in the risk of disease progression (HR=0.3; 95% CI: 0.1–0.7, p=0.004). No statistically significant difference in OS was observed between patients with and without irAEs (p=0.053).

**Figure 1:**
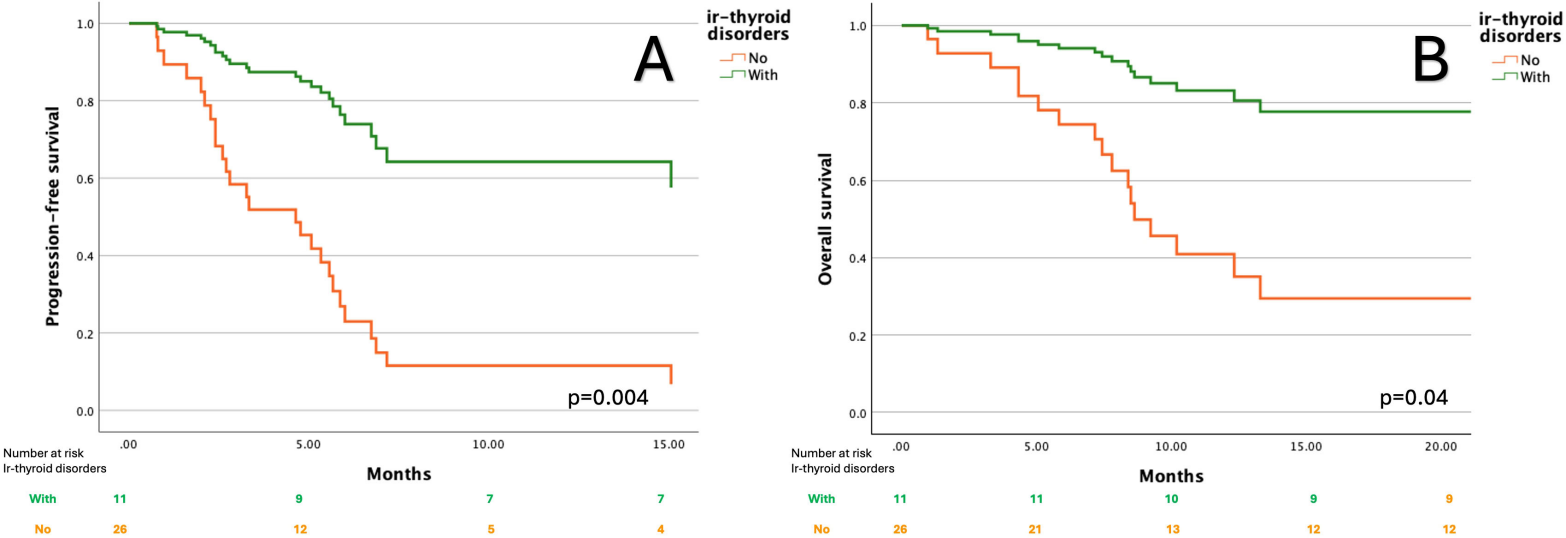
Kaplan–Meier curves for progression-free survival **(A)** and overall survival **(B)** according to the presence of immune-related thyroid disorders.

Among individual irAEs, ir-thyroid disorders were most strongly associated with favorable outcomes. Patients who developed ir-thyroid disorders had an 80% reduced risk of disease progression (HR =0.2; 95% CI: 0.07–0.6, p=0.004) and significantly longer PFS with median PFS NR (95% CI: NR–NR) compared to 4.6 months (95% CI: 2.4–6.8) in those without this toxicity (**Figure 1A**). Additionally, this subgroup had an 80% reduction in the risk of death (HR=0.2; 95% CI: 0.05–0.9, p=0.04). Median OS was NR in patients with ir-thyroid disorders (95% CI: NR–NR), whereas it was 8.6 months (95% CI: 7.4–9.8) in those without such events (**Figure 1B**). In a multivariate Cox proportional hazards model adjusting for clinically relevant potential confounders (age ≥65 years, ECOG performance status, line of therapy, and current FIGO stage), the occurrence of ir-thyroid disorders remained an independent predictor of improved PFS (HR=0.27; 95%CI: 0.07-0.96, p=0.04) and OS (HR=0.16; 95%CI: 0.03-0.8, p=0.02). Detailed results of this analysis are provided in **Supplementary Tables S1**, **S2**.

**Figure 2:**
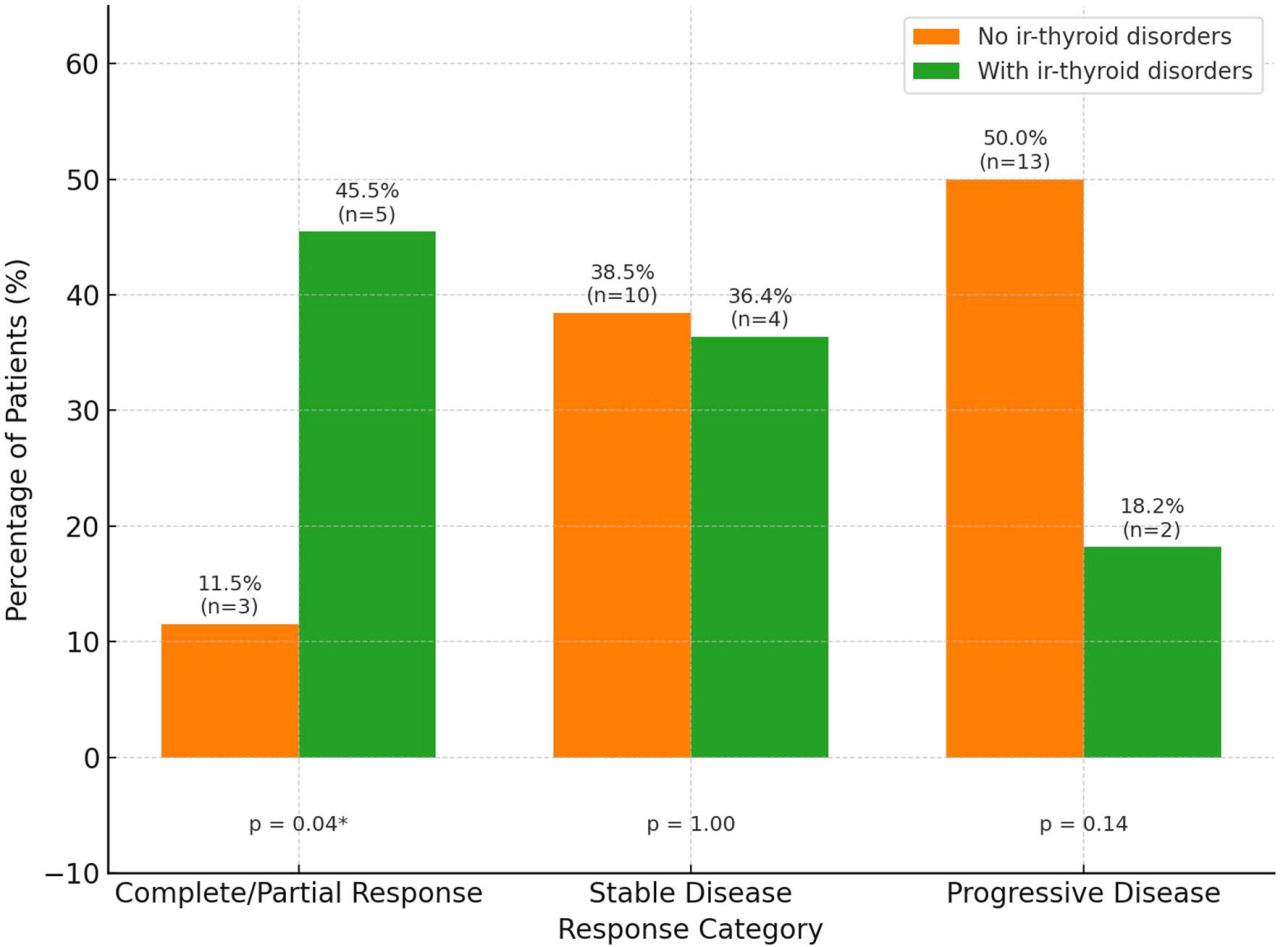
Treatment response by the presence of immune-related thyroid disorders (ir-thyroid disorders). Percentages and number of patients (n) are shown above each bar. Statistical significance is displayed below each response category. Asterisk * indicates p < 0.05.

A significantly higher proportion of patients with ir-thyroid disorders achieved objective responses compared to those without (45.5% vs. 11.5%, p=0.04), as shown in **Figure 2**. No similar associations were found in the case of other irAEs. The median time to ir-thyroid disorders onset was 2 months (interquartile ranges [IQR]: 1–3.5 months) from treatment initiation.

Non-immune-related AEs were also observed, most commonly anemia due to other causes (iron deficiency or anemia of chronic diseases) occurring in 21.6% of patients (n=8) and a single case of posterior reversible encephalopathy syndrome (PRES) secondary to uncontrolled hypertension (n=1, 2.7%).

### 3.3 Survival outcomes

Patients received a median of 6 cycles of cemiplimab (IQR: 4–8.75) over a median treatment duration of 5 months (IQR: 2.1–8). After a median follow-up of 9.2 months (IQR: 6.8–12.9), the median PFS was 5.6 months (95% CI: 4.3–6.8), while the median OS reached 12.3 months (95% CI: 8.1–17.6). An ORR of 21.6% (n=8) was observed, with stable disease (SD) in 37.8% (n=14) and progressive disease (PD) in 40.5% (n=15).

At the data cut-off, 9 patients (24.3%) remained on treatment, while 28 patients (75.7%) had discontinued therapy, primarily due to disease progression or death (n=27, 73.0%) and, in one case, due to treatment-related toxicity (n=1, 2.7%). A total of 18 patients (48.6%) had died by the end of the observation period, whereas 19 patients (51.4%) were alive at the time of data cut-off.

## 4 Discussion

These findings suggest that the development of irAEs may serve as a predictive marker of treatment efficacy in patients with r/mCC receiving cemiplimab. Among all irAEs, ir-thyroid disorders were the most frequently observed and demonstrated a particularly strong association with prolonged PFS and OS. The median time for all types of irAEs onset, as well as ir-thyroid disorders of approximately two months, highlights the importance of early and proactive toxicity monitoring during treatment.

Previous studies in other malignancies have reported associations between endocrine irAEs and favorable clinical outcomes. For instance, a retrospective analysis of 154 patients with metastatic melanoma treated with ipilimumab demonstrated that those who developed hypophysitis (n=17) had significantly improved survival outcomes compared to those without this toxicity (median OS: 19.4 vs. 8.8 months, p=0.05) (15). In a large multicenter analysis by Ashi et al. (16), patients experiencing endocrine toxicity had higher ORR to immunotherapy (45% vs. 28%, p<0.001), which translated into significantly longer OS. A similar trend was observed in patients with non-small cell lung cancer, where the presence of ir-thyroid disorders was associated with numerically longer survival, although the difference did not reach statistical significance (17). These findings of improved OS and PFS in patients with ir-thyroid disorders during ICIs therapy were further confirmed by Basak et al. (18) and Lu et al. (19) in patients with various malignancies.

**Figure 3:**
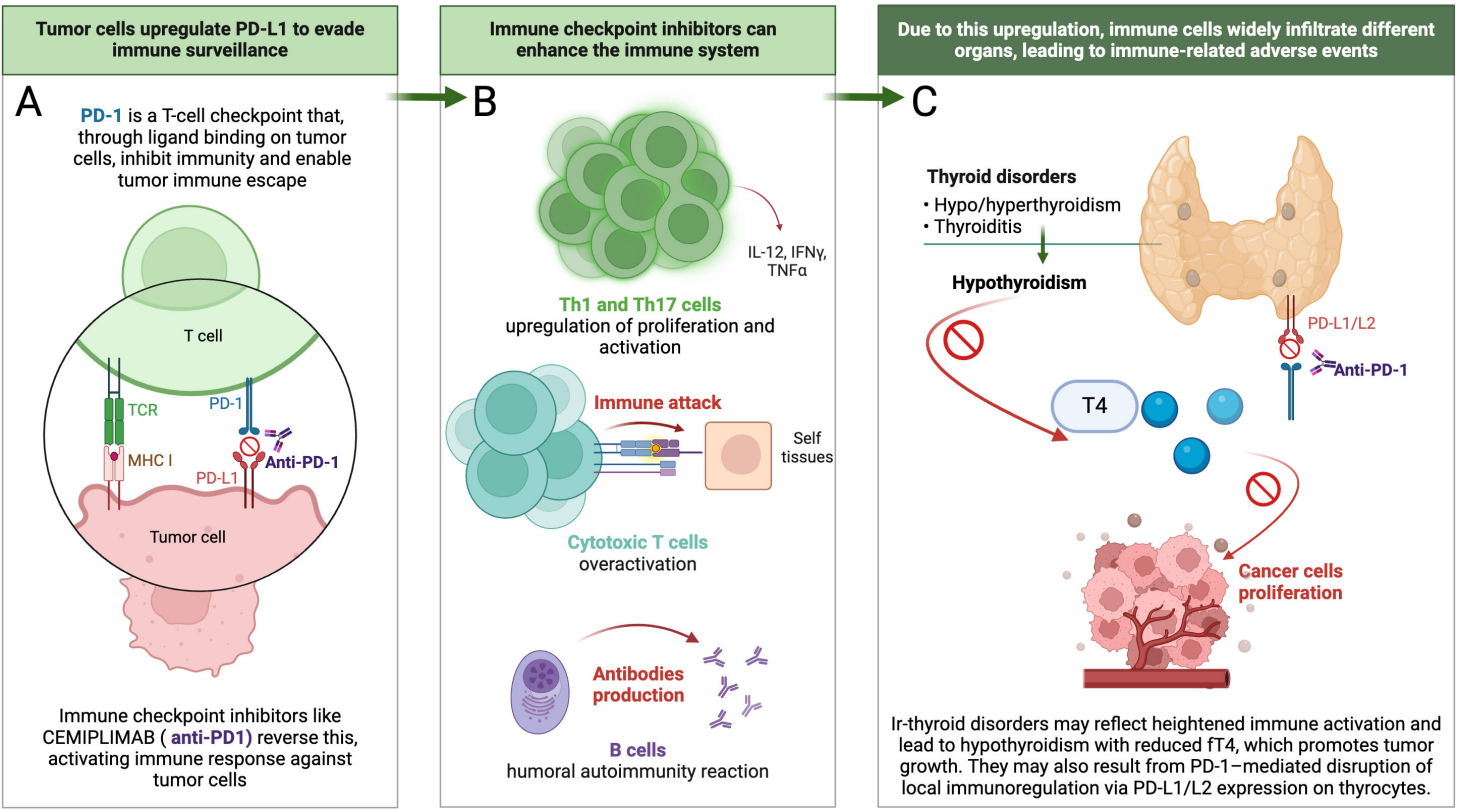
Proposed sequence of events linking cemiplimab-induced ir- thyroid disorders with improved clinical outcomes. **(A)** Mechanism of action of cemiplimab at the immunological synapse, illustrating PD-1 blockade and reactivation of tumor-specific T cells; **(B)** Cascade of immune activation following ICIs therapy, leading to enhanced effector T-cell and B-cell responses; **(C)** Development of ir-thyroid disorders, ultimately resulting in hypothyroidism and reduced circulating T4 levels, a hormone known to promote tumor growth and angiogenesis.

The association between irAEs and improved clinical outcomes in patients receiving ICIs is likely multifactorial and driven by enhanced immune activation. Two main immunopathogenic mechanisms have been proposed to explain the development of irAEs. First, ICI-induced T-cell hyperactivation amplifies antitumor immunity but can also cause “on-target” toxicity by attacking normal tissues that share antigenic similarity with tumor cells. This effect may be further exacerbated by epitope spreading, in which antigens released from lysed tumor cells expand the T-cell repertoire and reduce immune tolerance by activating T cells against corresponding self-antigens expressed in healthy tissues. Second, ICIs therapy promotes polarization toward Th1 and Th17 phenotypes, resulting in elevated levels of proinflammatory cytokines such as Interferon-gamma (IFN-γ) and Interleukin-17 (IL-17), which support antitumor activity but also contribute to systemic inflammation and “off-target” irAEs (20–22). In addition, anti-PD-1 therapy has been shown to disrupt peripheral tolerance by removing inhibitory signals from CD8+ T cells, leading to their unchecked expansion and acquisition of autoreactive cytotoxic functions. These CD8+ T cells can damage normal tissues through direct cytolytic mechanisms, as demonstrated by dense CD8+ infiltration in affected tissues (22). B-cell involvement has also been implicated in irAEs pathogenesis. Autoantibody production is frequently observed in affected patients, and some individuals exhibit low-titer autoantibodies even before the clinical presentation of irAEs. Their subsequent rise during treatment and association with irAEs development suggest a breakdown of peripheral tolerance of preexisting self-reactive clones (20, 23). Monitoring autoantibodies before and during treatment may help identify patients at increased risk of irAEs and holds promise for the development of predictive biomarkers. However, data remain inconclusive. In the study by Lu et al. (19), baseline levels of anti-thyroid antibodies (e.g., thyroid peroxidase and thyroglobulin antibodies) were not associated with the development of thyroid dysfunction during anti-PD-1 therapy. In contrast, other studies have shown that patients who exhibited a significant rise in antibody titers during treatment had improved survival outcomes (18), suggesting a potential prognostic role for treatment-induced autoantibody dynamics. In addition to systemic immune activation, ir-thyroid disorders may arise from constitutive PD-L1/PD-L2 expression on thyrocytes. Blockade of PD-1 interferes with this local immunoregulatory axis, potentially allowing cytotoxic T-cell infiltration and tissue damage, even without prior autoimmunity or antigenic mimicry (24). These events are summarized in **Figure 3B**.

Hypothyroidism—whether spontaneous or induced—has been shown to influence tumor biology across various malignancies. One particularly intriguing therapeutic approach involves pharmacologically induced hypothyroidism using propylthiouracil, followed by high-dose tamoxifen administration in patients with recurrent high-grade glioma. This strategy has been associated with significantly prolonged survival (10.1 vs. 3.1 months, p=0.03), alongside a reduction in circulating insulin-like growth factor 1 levels (25). In a cohort study of 1,136 women, those with a diagnosis of primary, symptomatic hypothyroidism receiving thyroid hormone replacement therapy exhibited a 61% reduced risk of developing invasive breast cancer (26). In patients with renal cell carcinoma, hypothyroidism, thyroid dysfunction in general, and the use of thyroid hormone supplementation were significantly more prevalent compared to controls of women with transitional cell carcinoma of the renal pelvis, ureter, bladder, or urethra (27). Moreover, experimental endocrine strategies such as maintaining a euthyroid hypothyroxinemic state—by administering T3 without T4—have been associated with slower disease progression in advanced malignancies (28).

Several mechanisms may underlie the observed associations between ir-thyroid disorders and cancer outcomes. One proposed explanation involves the identification of a specific receptor for thyroid hormone analogues located on the plasma membrane of malignant and rapidly proliferating endothelial cells (29). Thyroid hormones exert non-genomic effects via integrin αvβ3, a receptor broadly expressed on tumor cells, promote PD-L1 and β-catenin expression in tumor cells through extracellular signal-regulated kinases 1 and 2 (ERK1/2), phosphoinositide 3-Kinase (PI3K), and signal transducer and activator of transcription 3 (STAT3)-dependent pathways, contributing to immune evasion and tumor progression (**Figure 3C**) (30). In contrast, T3, although potentially proliferative at high concentrations, does not appear to stimulate tumor growth when used alone to maintain a euthyroid state, suggesting a meaningful therapeutic distinction between T3 and T4 (31).

While ir-thyroid disorders were significantly associated with improved outcomes, other irAEs did not demonstrate similar correlations in this study. This may be attributed to the limited number of events for non-thyroid irAEs, reducing statistical power. Additionally, thyroid dysfunctions are often driven by the abovementioned mechanisms that may more closely reflect effective systemic immune activation. In contrast, other irAEs, such as hepatic toxicity or infusion reactions, may arise from divergent or nonspecific immune pathways that are not directly linked to antitumor efficacy (20–22).

In summary, the improved prognosis observed in patients who developed ir-thyroid disorders during cemiplimab treatment reflects a more robust immune system activation. Their occurrence may indicate heightened immunologic responsiveness not only against endocrine self-antigens but also against tumor-associated antigens, thereby translating into improved tumor control. The pathophysiological basis of this association likely involves immune dysregulation, loss of peripheral tolerance, and disruption of the PD-1/PD-L1 axis in thyroid tissue, which may reflect a broader systemic antitumor immune response. Furthermore, all three clinical manifestations of ir-thyroid disorders—primary hypothyroidism, hyperthyroidism, and thyroiditis—tend to culminate in hypothyroidism accompanied by decreased T4 levels, which has been implicated in promoting tumor cell proliferation. This sequence of events is illustrated in **Figure 3**. However, given the small sample size and exploratory nature of this real-world analysis, these findings should be interpreted with caution. Larger-scale, prospective studies are warranted to validate the predictive value of ir-thyroid disorders.

Our study has several limitations. The relatively small sample size and restricted patient access, limited to reference centers participating in the rescue access program, may introduce selection bias and limit the generalizability of the findings to the broader r/mCC population. Nevertheless, within the constraints of the program, the study cohort was representative of real-world clinical practice, encompassing a range of histologic subtypes and ECOG performance statuses (0–2). All patients were treated in Poland, which may not fully reflect patient characteristics and treatment accessibility, observed in other healthcare systems or ethnic groups. Therefore, our findings should be interpreted in the context of a Central European population and warrant validation in more diverse, multinational cohorts. Furthermore, the median follow-up time of 9.2 months may limit the reliability of long-term survival estimates, but only a minority of patients were still on treatment at the time of data cut-off. Additionally, imaging and biomarker assessments (PD-L1, CPS) were performed locally, without central review, which may have introduced variability in response classification. Safety data were collected from medical records, potentially underestimating irAEs compared to prospective clinical trials. As this was a retrospective study, thyroid-specific diagnostics, including uptake tests, imaging, antibody panels, and thyroglobulin measurements, were not routinely performed, as most patients were not initially under endocrinological care. The limitations of our ambispective real-world design—including potential selection bias, information bias, and residual confounding—are acknowledged and discussed in detail in the **Supplementary Materials**. These factors may have affected the strength of associations observed in this preliminary cohort and should be considered when interpreting the results.

## 5 Conclusion

This real-world study suggests that irAEs—particularly ir-thyroid disorders—may serve as potential predictive biomarkers of cemiplimab efficacy in r/mCC, with observed associations for both PFS and OS. These findings warrant further validation in larger, prospectively collected cohorts and through the integration of extended immunological profiling, including autoantibody panels or dynamic monitoring of thyroid function.

## Data Availability

The datasets used and/or analysed during the current study are available from the corresponding author upon reasonable request.
