# Supplementary material for: Thyroid disorders as predictors of cemiplimab efficacy in recurrent/metastatic cervical cancer: real-world evidence from Poland
Source: Front Immunol. 2025 Jun 20;16:1604826. doi: 10.3389/fimmu.2025.1604826 (PMC12226472; doi:10.3389/fimmu.2025.1604826)
Supplement: Supplementary file 1 [file Table1.docx]

Supplementary Material

1. **Detailed methods description based on the European Society for Medical Oncology (ESMO) Good Reporting of Outcomes in Real-World Evidence Studies (GROW)** [1] **guidelines.**
   1. **Study research question**

The primary research question of this study was to evaluate whether the occurrence of immune-related adverse events (irAEs) is associated with improved clinical outcomes in patients with recurrent or metastatic cervical cancer (r/mCC) treated with cemiplimab in a real-world setting. This analysis aimed to generate real-world evidence on the potential role of irAEs as biomarkers of response to anti-programmed death-1 (PD-1) immunotherapy in this patient population.

- 1. Study design and adherence to guidelines

This was an ambispective, multicenter, real-world study. Baseline demographic and clinical characteristics were collected retrospectively from electronic health records (EHRs), while treatment outcomes and safety data were assessed prospectively during routine clinical follow-up. The study methodology was developed following the European Society for Medical Oncology (ESMO) Good Reporting of Outcomes in Real-World Evidence (GROW) recommendations [1]. Adverse events (AEs), including immune-related adverse events (irAEs), were identified, graded, and reported based on the Common Terminology Criteria for Adverse Events (CTCAE) version 5.0 [2]. The definition, classification, and management of irAEs followed ESMO Clinical Practice Guidelines [3]. Tumor response was evaluated locally at each participating center by dedicated oncologists and radiologists according to the Response Evaluation Criteria in Solid Tumors version 1.1 (RECIST v1.1) guidelines [4]. Radiological assessments were performed using computed tomography (CT) scans of the chest, abdomen, and pelvis at predefined intervals every 12 weeks or earlier if clinically indicated.

- 1. Data sources, data management and core variables

This study was based on data obtained from EHRs of patients with r/mCC treated with cemiplimab. The EHRs were considered appropriate for the study objectives due to their comprehensive capture of patient demographics, clinical characteristics, biomarker data, treatment exposures, therapeutic outcomes, and AEs.

- 1. Data origin and management

Data collection covered patients treated between October 1, 2022 and January 31, 2025, with the final data cut-off on March 1, 2025. Data extraction was conducted retrospectively by trained clinical researchers using a standardized electronic data collection template. Completeness and consistency were verified by cross-referencing extracted data with original EHR entries. Patient data were pseudonymized by assigning unique codes based on patient initials, ensuring confidentiality throughout the research process. No external audits or independent validation were performed.

The database was developed and governed by the coordinating center under the supervision of the first/corresponding author. Data ownership rests with the participating centers. Due to the sensitive nature of the data, the dataset is not publicly accessible but may be shared upon reasonable request and following appropriate ethical approvals. No external database registration or metadata repository applies.

1.4.1. Data linkage and duplication

All data originated from individual participating centers. To prevent duplicate entries, patients were uniquely identified by coded initials. No linkage with external databases or registries was performed.

- - 1. Core variables and definitions

The following core variables were collected and categorized:

- Baseline characteristics: Age at cemiplimab initiation, Eastern Cooperative Oncology Group (ECOG) performance status, International Federation of Gynecology and Obstetrics (FIGO) stage at diagnosis, histological subtype, Human papillomavirus (HPV) status (when available), and PD-L1 expression. PD-L1 testing was performed on formalin-fixed, paraffin-embedded tumor specimens using the Monoclonal Mouse Anti-Human PD-L1 antibody (DAKO) and the EnVision Flex High pH Detection Kit (DAKO). PD-L1 expression was quantified as the Combined Positive Score (CPS, defined below).
- Exposure**:** Cemiplimab administration (start date, dosing schedule, and treatment duration).
- Outcomes/Endpoints:

**Primary objective-** to assess the safety of cemiplimab by evaluating:

- - Incidence, type, and severity of irAEs
  - Grading of irAEs according to CTCAE v5.0 [2]
  - Association of irAEs with survival outcomes: PFS and OS

**Secondary objective-** to evaluate treatment efficacy, including:

- - Progression-free survival (PFS)
  - Overall survival (OS)
  - Overall response rate (ORR)
  - Disease control rate (DCR)
  - Tumor response assessed by CT scans (chest, abdomen, pelvis) every 12 weeks or earlier if clinically indicated according to RECIST v1.1 criteria [4].
    1. Handling missing data

Data completeness was verified during the extraction process. In cases of missing values, no imputation methods were applied. Analyses were performed using a complete case approach, excluding patients with missing data for the variables relevant to each specific analysis

- 1. Bias and confounding
     1. This study is subject to several potential sources of bias and confounding, inherent to its real-world and ambispective design, including retrospective data collection components.
- **Information bias**
  – Resulting from the retrospective nature of data collection, which depended on the accuracy and completeness of clinical records.
  – Particularly relevant for underreporting or misclassification of low-grade or subclinical irAEs, especially thyroid dysfunction.
  – Additional variability was introduced by the lack of centralized pathological review; PD-L1 (CPS) and HPV testing were performed locally at each center.
- **Selection bias**
  – Arising from physician-driven patient inclusion into the rescue access program, with external validation by regional authorities.
  – May have favoured patients with better performance status or fewer comorbidities, limiting generalizability.
- **Residual confounding**
  – Potentially related to unmeasured clinical factors (e.g., tumor burden, use of steroids) influencing both irAEs development and treatment outcomes.
  – Full adjustment for these variables was not possible due to limitations in available data.
- **Immortal time bias**
  – Patients must survive a minimum duration to develop irAEs, which may artificially inflate survival in the irAEs-positive group.
- **Reporting bias**
  – Especially relevant for mild adverse events that may not have been documented unless clinically significant or requiring intervention.
- **Interobserver and intercenter variability**
  – Differences in clinical judgment, diagnostic intensity (e.g., frequency of thyroid testing), and response assessment across centers without central adjudication may have influenced irAEs detection and outcome evaluation.

**1.5.2. Strategies to Mitigate Bias:**

- To reduce potential sources of bias, predefined case definitions were applied consistently across all participating centers, and data underwent rigorous manual validation. Extracted variables were cross-checked against original electronic health records, and any discrepancies were clarified directly with treating physicians. Multicenter design contributed to external validity, while efforts to standardize data collection minimized interobserver variability. Importantly, the study adhered to established methodological standards, including the ESMO guidelines for irAEs management [3], the CTCAE v5.0 [2] for toxicity grading, and the RECIST v1.1 [4] for radiologic assessment.

1. **Definitions**

2.1. Efficacy parameters

- Overall survival (OS) - the duration from the initiation of immunotherapy to the patient's death
- Progression-free survival (PFS)- the period from the onset of immunotherapy to progressive disease (PD) on CT scan or patient death due to any cause
- Overall response rate (ORR), comprising complete remission (CR) or partial response (PR)
- Disease control rate (DCR), encompassing CR, PR, and stable disease (SD) (both according to RECIST 1.1 [5]

2.3. Thyroid toxicities [6]

- Primary hypothyroidism- a free thyroxine (T4) level below the lower limit of normal with an elevated thyroid-stimulating hormone (TSH) level
- Primary hyperthyroidism - an increased free T4 or 3,3′,5-triiodo-L-thyronine (T3) levels accompanied by a suppressed TSH level
- Thyroiditis was identified by an initial thyrotoxicosis phase followed by spontaneous hypothyroidism

2.3. Other terms

- Combined positive score (CPS)- defined as the ratio of PD-L1-positive cells (tumor cells, lymphocytes, macrophages) to the total number of viable tumor cells multiplied by 100. PD-L1 positivity was defined as CPS >1.

1. **Multivariate Cox regression models for progression-free survival and overall survival with factors identified as clinically relevant potential confounders.**

Table S1. Multivariate Cox regression model for progression-free survival (p for the model 0.3).

|  | Hazard ratio | 95% CI | p-value |
| --- | --- | --- | --- |
| Ir-thyroid disorders | 0.27 | 0.07-0.96 | 0.04* |
| Age≥65 years | 0.99 | 0.38-2.6 | 1 |
| ECOG performance status | 1.42 | 0.59-3.42 | 0.44 |
| Histology | 1.54 | 0.52-4.6 | 0.44 |
| Line of treatment | 0.97 | 0.49-1.92 | 0.93 |
| Current FIGO stage | 1.31 | 0.34-5.14 | 0.7 |

Abbreviations: CI- Confidence interval; ECOG- Eastern Cooperative Oncology Group; Ir- immune-related; FIGO- International Federation of Gynecology and Obstetrics;
Values with statistical significance are marked as *.

Table S2. Multivariate Cox regression model for overall survival (p for the model 0.11).

|  | Hazard ratio | 95% CI | p-value |
| --- | --- | --- | --- |
| Ir-thyroid disorders | 0.16 | 0.03-0.77 | 0.02* |
| Age≥65 years | 0.75 | 0.24-2.35 | 0.63 |
| ECOG performance status | 2.94 | 1.01-8.56 | 0.047* |
| Histology | 0.65 | 0.18-2.34 | 0.5 |
| Line of treatment | 1.06 | 0.5-2.23 | 0.88 |
| Current FIGO stage | 0.84 | 0.16-4.47 | 0.84 |

Abbreviations: CI- Confidence interval; ECOG- Eastern Cooperative Oncology Group; Ir- immune-related; FIGO- International Federation of Gynecology and Obstetrics;
Values with statistical significance are marked as *.

**4. References**

1. Castelo-Branco L, Pellat A, Martins-Branco D, et al (2023) ESMO Guidance for Reporting Oncology real-World evidence (GROW). Annals of Oncology 34:1097–1112

2. Common Terminology Criteria for Adverse Events (CTCAE) v5.0. https://ctep.cancer.gov/protocoldevelopment/electronic_applications/docs/CTCAE_v5_Quick_Reference_5x7.pdf. Accessed 21 Jan 2025

3. Haanen J, Obeid M, Spain L, et al (2022) Management of toxicities from immunotherapy: ESMO Clinical Practice Guideline for diagnosis, treatment and follow-up 5 behalf of the ESMO Guidelines Committee. 33:1217–1238

4. Schwartz LH, Litière S, De Vries E, et al (2016) RECIST 1.1-Update and clarification: From the RECIST committee. Eur J Cancer 62:132–137

5. Somarouthu B, Lee SI, Urban T, Sadow CA, Harris GJ, Kambadakone A (2018) Immune-related tumour response assessment criteria: a comprehensive review. Br J Radiol 91:736

6. Podręcznik Interna. https://www.mp.pl/interna/. Accessed 7 Aug 2024
